# Mechanically assisted non-invasive ventilation for liver SABR: Improve CBCT, treat more accurately

**DOI:** 10.1016/j.ctro.2025.100983

**Published:** 2025-05-22

**Authors:** Julien Pierrard, Nicolas Audag, Christel Abdel Massih, Maria Alvear Garcia, Enrique Alvarez Moreno, Andrea Colot, Simon Jardinet, Romain Mony, Ana Francisca Nevez Marques, Lola Servaes, Thaïs Tison, Valentin Van den Bossche, Aniko Wale Etume, Lamyae Zouheir, Geneviève Van Ooteghem

**Affiliations:** a Institut de Recherche Experimentale et Clinique (IREC), Center of Molecular Imaging, Radiotherapy and Oncology (MIRO), Université Catholique de Louvain, Brussels, Belgium; b Department of Radiation Oncology, Cliniques Universitaires Saint-Luc, Brussels, Belgium; c Institut de Recherche Expérimentale et Clinique (IREC), Pôle de Pneumologie, ORL (airways) & Dermatologie (skin), Groupe Recherche en Kinésithérapie Respiratoire, Université Catholique de Louvain, Brussels, Belgium; d Service de kinésithérapie et ergothérapie, Cliniques universitaires Saint-Luc, Brussels, Belgium

**Keywords:** Image-guided radiotherapy, Liver SABR, Mechanically assisted non-invasive ventilation, Respiratory motion management, CBCT quality

## Abstract

**Background and purpose:**

Cone-beam computed tomography (CBCT) for image-guided radiotherapy (IGRT) during liver stereotactic ablative radiotherapy (SABR) is degraded by respiratory motion artefacts, potentially jeopardising treatment accuracy. Mechanically assisted non-invasive ventilation-induced breath-hold (MANIV-BH) can reduce these artefacts. This study compares MANIV-BH and free-breathing CBCTs regarding image quality, IGRT variability, automatic registration accuracy, and deep-learning auto-segmentation performance.

**Materials and methods:**

Liver SABR CBCTs were presented blindly to 14 operators: 25 patients with FB and 25 with MANIV-BH. They rated CBCT quality and IGRT ease (rigid registration with planning CT). Interoperator IGRT variability was compared between FB and MANIV-BH. Automatic gross tumour volume (GTV) mapping accuracy was compared using automatic rigid registration and image-guided deformable registration. Deep-learning organ-at-risk (OAR) auto-segmentation was rated by an operator, who recorded the time dedicated for manual correction of these volumes.

**Results:**

MANIV-BH significantly improved CBCT image quality (“Excellent”/“Good”: 83.4 % versus 25.4 % with FB, p < 0.001), facilitated IGRT (“Very easy”/“Easy”: 68.0 % versus 38.9 % with FB, p < 0.001), and reduced IGRT variability, particularly for trained operators (overall variability of 3.2 mm versus 4.6 mm with FB, p = 0.010). MANIV-BH improved deep-learning auto-segmentation performance (80.0 % rated “Excellent”/“Good” versus 4.0 % with FB, p < 0.001), and reduced median manual correction time by 54.2 % compared to FB (p < 0.001). However, automatic GTV mapping accuracy was not significantly different between MANIV-BH and FB.

**Conclusion:**

In liver SABR, MANIV-BH significantly improves CBCT quality, reduces interoperator IGRT variability, and enhances OAR auto-segmentation. Beyond being safe and effective for respiratory motion mitigation, MANIV increases accuracy during treatment delivery, although its implementation requires resources.

## Highlights

• Mechanically assisted non-invasive ventilation is used for CBCT-guided liver SABR.
• CBCT image quality is better with MANIV compared to free-breathing.
• MANIV facilitates IGRT and reduces its interobserver variability.
• OARs auto-segmentation is more accurate on MANIV CBCTs.
• Time dedicated to correction of auto-segmented OARs is reduced with MANIV CBCTs.

## 1 Introduction

Liver tumours, including primary malignancies and metastases, represent a significant global health issue due to rising incidence and persistently high mortality rates [[1], [2], [3]]. Liver stereotactic ablative radiotherapy (SABR) is increasingly used for managing both primary and secondary tumours [[4], [5], [6]]. In curative treatment of hepatocellular carcinoma and colorectal liver metastases, SABR can serve as a bridging therapy to enhance local control before transplantation [4,5]. For patients ineligible for transplantation or surgery, SABR offers a safe and effective alternative to radiofrequency ablation and transarterial chemoembolization, as recommended by ESMO and EASL guidelines [4,[7], [8], [9]]. SABR achieves up to 82 % 5-year local control in hepatocellular carcinoma [6]. However, accurate image-guided radiotherapy (IGRT) and effective respiratory motion management remain critical challenges for accurate targeting and sparing of organs-at-risk (OARs).

Accurate IGRT is complex due to the similar density of liver tumours and surrounding tissue on cone-beam computed tomography (CBCT), with image quality often compromised by respiratory artefacts [10]. Strategies to overcome this include magnetic resonance (MR)-guided RT, which enables direct and real-time visualisation of liver tumours and adjacent OARs during daily IGRT [[11], [12], [13]]. Another option is the percutaneous implantation of radiopaque markers as surrogates for tumour position during CBCT-guided IGRT [14]. Recently, CBCT-guided online-adaptive RT (oART) has been evaluated for liver SABR [15]. In oART, OARs are automatically delineated, and target volumes are automatically propagated from planning CT to daily CBCT using a deformable registration. While adaptive planning can improve OARs sparing and target volume coverage, automatic propagation of target volumes introduces uncertainties that should be integrated in planning target volume (PTV) margins. In the context of liver SABR, these uncertainties results in PTV margins that are larger in oART compared to non-ART [15]. In practice, many centres still avoid such complex, resource-intensive techniques and rely on broader margins despite their associated limitations.

Respiratory motion strategies include passive (e.g., internal target volume, mid-position, abdominal compression) or active techniques (e.g., breath-hold, respiratory gating, tumour tracking) [16]. Passive techniques are widely used due to ease of implementation. Active strategies, though more complex and resource demanding, aim for greater accuracy with reduced OAR irradiation. Among them, mechanically-assisted non-invasive ventilation (MANIV) for breath-hold (BH) is particularly promising. MANIV-BH enables reproducible deep inspiration breath-holds, reducing breathing-related motion [[17], [18], [19]]. MRI studies have confirmed its feasibility and ability to induce stable apneas [[17], [18], [19], [20]]. MANIV-BH is safe for liver SABR and, compared to other techniques, may reduce PTV margin [19]. Moreover, immobilisation of the thorax and upper abdomen during MANIV-BH could be can minimise respiratory artefacts on CBCT and thereby improving IGRT accuracy.

This study aims to compare CBCTs acquired with MANIV-BH versus free-breathing (FB) CBCTs in liver SABR patients to assess their impact on image quality and treatment precision. The impact of MANIV-BH was assessed through a multi-step approach for both non-adaptive RT (Non-ART) and oART strategies, progressing from basic to advanced metrics: (1) Image quality assessment; (2) IGRT variability; (3) Automatic registration accuracy; and (4) deep-learning auto-segmentation performance.

## 2 Materials and methods

This monocentric *in silico* study was conducted in accordance with the Declaration of Helsinki and approved by the ethics committee of Cliniques Universitaires Saint-Luc.

### 2.1 Patients

Patient were retrospectively included between January 2021 and July 2024 and allocated into two groups based on the respiratory motion management strategy: MANIV-BH versus FB. The sample size was fixed a priori at 25 patients per group. Inclusion criteria were as follows: (1) adult patients (>18 years old); (2) patients treated with liver SABR or RT for stomach, adrenal or pancreas tumour that included an identifiable structure within the liver (e.g., cyst, hemangioma or tumour) on planning CT; (3) patients treated on linear accelerators sharing identical CBCT acquisition device (Halcyon® and Ethos®, Varian, a Siemens Healthineers Company, Palo Alto, CA).

### 2.2 RT treatment and CBCT acquisition

**Fig. 1:**
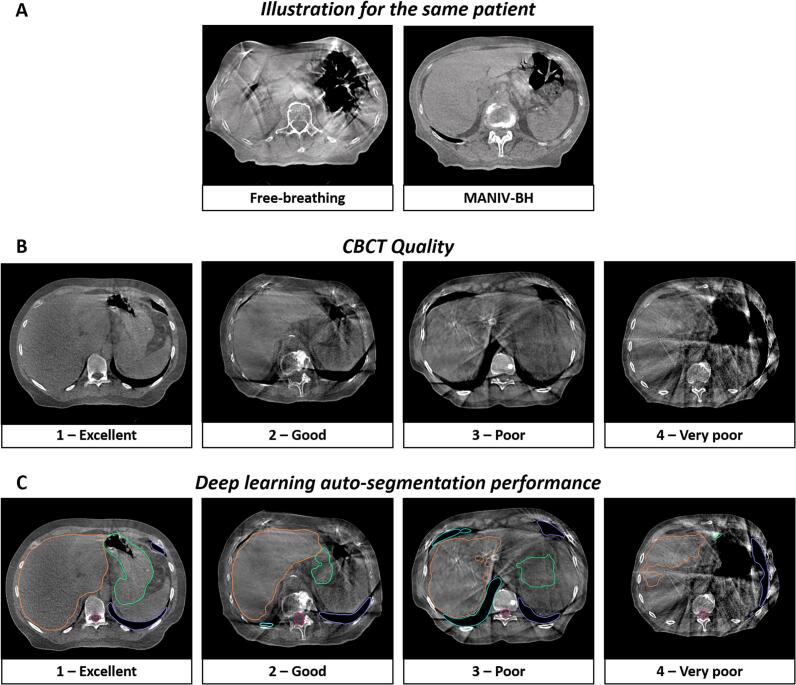
Illustrations of (A) CBCTs acquired with free-breathing and with MANIV-BH for the same patient, (B) different CBCT quality and (C) performance of auto-segmentation by a deep learning algorithm. (Should NOT be printed in colours) CBCT: Cone-beam computed tomography, MANIV-BH: Mechanically-assisted non-invasive ventilation for breath-old.

For FB patients, a 4D planning CT with audio-coaching and abdominal compression was acquired, while for MANIV-BH patients a 3D planning CT was used. All CTs were acquired in supine with intravenous iodine contrast injection. RT planning was performed using the Raystation Planning system (clinical versions 12A, RaySearch Laboratories, Stockholm, Sweden). Prescription doses and target volume delineation were adapted based on the RT intent. Daily CBCTs were acquired using the “pelvis fast” (acquisition time: 21.2 s) or “pelvis large fast” (acquisition time: 25 s) modes on both linear accelerators for FB and MANIV-BH patients (slice thickness: 2 mm, Fig. 1A). IGRT was performed using a tumour-based rigid registration between the planning CT and the CBCTs. For MANIV-BH patients, multiple CBCTs were acquired to assess the BH reproducibility (internal IGRT protocol).

MANIV-BH procedure has been described in detail in previous studies (Fig. 1A) [17,19]. Briefly, prior to the planning CT acquisition, patients were trained to familiarise with the procedure. The ventilatory parameters were individually determined by a physiotherapist. Following connexion to the mechanical ventilator (Bellavista 1000, Vyaire Medical, Mettawa, Illinois, USA), pre-oxygenation was initiated (Fi60%) while patients continued to breathe without constraint. To induce apnea, the adaptive pressure release ventilation mode was activated. This mode consists of two phases: (1) Inhale and apnea phase: a high constant pressure (range: 15–19 mmHg) was sustained for 30 s; (2) Exhale phase: a zero-pressure state (range: 4.1–5.1 s) allowed the patient to breathe out. This cycle (1 + 2) was repeated throughout the RT session. The radiation therapy technologist (RTT) manually activated the beam during the apneas and interrupted the beam just before the second phase, based on the ventilator’s pressure curve, which was displayed in the control room.

For this study, the planning CT with the gross tumour volume (GTV) of the liver and the first CBCT of each of the 50 included patients were used. In cases of pancreatic, adrenal or stomach cancer treatment, a “virtual” liver GTV was retrospectively delineated based on a pre-existing identifiable structure selected within the liver. All CBCTs were anonymised and presented randomly and blindly to the participating operators.

### 2.3 Image quality assessment

Nine radiation oncologists (ROs) and five RTTs assessed the quality of CBCT images using a four-point scoring scale developed for this study: 1 – Excellent, 2 – Good, 3 – Poor, and 4 – Very poor.

### 2.4 IGRT variability

The same operators then performed a GTV-based rigid registration between the planning CT and the CBCTs using translation only, mimicking the actual clinical practice in the institution. They rated the ease of performing this matching using another four-point scoring scale developed for this study: 1 – Very easy, 2 – Easy, 3 – Hard, and 4 – Very hard. For each CBCT, the standard deviation of the registration vector across operators was calculated in right-left, antero-posterior and supero-inferior directions. The overall variability was quantified using the total standard deviation of the registration, defined as the square root of the sum of the variances in each dimension. Among the ROs, five had over four years of experience and were considered fully trained in liver SABR IGRT. A separate analysis of this subgroup was conducted to provide a more accurate reflection of clinical practice.

### 2.5 Automatic GTV mapping

Two automatic GTV mappings from the planning CT to the CBCT were analysed, corresponding to conventional Non-ART and oART strategies.

For the Non-ART strategy, an automatic rigid registration using only translations focused on the GTV region of interest was performed between the planning CT and the CBCT using the Raystation® Planning system. The GTV from the planning CT was then transferred to the CBCT (GTV_Non-ART_).

For the oART strategy, virtual oART sessions were conducted using the Ethos® emulator. This emulator includes both the oART treatment planning system and the online adaptive radiotherapy software, enabling virtual delivery of oART sessions using planning CT and CBCT images retrieved from a database. During these virtual sessions, the GTV was automatically propagated (GTV_oART_) onto the CBCT via image-guided deformable registration with rigid volume propagation (research version). However, the emulator was no longer provided to our department by the vendor at the time of this analysis, and results were only available for the first 20 MANIV-BH patients. These results were compared to a previously published cohort of 21 FB patients [15].

The methodology used for propagation accuracy assessment was detailed in prior work [15]. Briefly, the propagated GTVs (GTV_Non-ART_ or GTV_oART_) were compared to the reference GTV positions on the daily CBCT (GTV_Groundtruth_). This GTV_Groundtruth_ was defined as the median GTV position among trained operators during the IGRT variability analysis. GTV positions were compared using two metrics: (1) the Euclidean distance between the centres of mass of the propagated GTV and the GTV_Groundtruth_ (CoM), and the Hausdorff 95 % distance, which is the greatest distance between these volumes among their 95 % closest voxels.

### 2.6 Deep-learning auto-segmentation performance

As for the previous analysis on “Automatic GTV mapping”, the availability of the ETHOS® emulator was discontinued for this analysis. Therefore, the deep-learning segmentation model integrated into the institutional treatment planning system (Raystation®) was used to delineate abdominal OAR volumes on each CBCT. A single RO assessed the segmentation quality for both overall and individual OARs using a four-point scoring scale: 1 – Excellent, 2 – Good, 3 – Poor, and 4 – Very poor. Subsequently, the RO manually corrected the segmented volumes on transversal CBCT slices over a 10 cm height, with the most cranial part of the L1 vertebral body serving as the central reference slice. The list of OARs included both lungs, the heart, the spinal canal, the liver, the oesophagus, the stomach, and both kidneys. The time required for manual corrections was recorded as an additional measure of auto-segmentation performance.

### 2.7 Statistical analysis

The median value and interquartile range (IQR) were used to describe quantitative data. Comparisons between FB and MANIV-BH groups were conducted using the Chi-square test for qualitative variables and the Wilcoxon signed-rank test for quantitative variables. A significance threshold was set at a p < 0.05. For multiple comparisons, p-values were adjusted using the Benjamini-Hochberg method to control for false discovery rates. All statistical analyses were performed using RStudio (R version 4.2.1) with the “tidyverse” package.

## 3 Results

The characteristics of the included patients are described in Table 1. For details on the FB population used in the “oART GTV propagation” analysis, refer to Supplementary Table 1.

**Table 1:** Characteristics of the patients.

	**FB patients**	**MANIV-BH patients**
**N**	25	25
**Gender**		
Male	15	15
Female	10	10
**Age (median [IQR])**	72 (66–79)	65 (56–72)
**Radiotherapy intent**		
*Primary disease*		
Hepatocarcinoma	12	9
Gastric lymphoma*	0	6
Pancreatic adenocarcinoma*	2	5
*Liver metastasis from*		
Colorectal adenocarcinoma	7	1
Non-small cell lung cancer	2	2
Breast cancer	1	0
Renal cell carcinoma	1	0
Cervical cancer	0	1
Gastroesophageal junction cancer	0	1
Tumour location**		
Segment II	2	4
Segment III	2	2
Segment IV	3	7
Segment V	5	4
Segment VI	6	4
Segment VII	6	4
Segment VIII	6	6
**GTV volume (cm^3^, median [IQR])**	9.2 (5.2–12.4)	8.8 (3.5–26.2)

*Patients for whom a “virtual” liver GTV was delineated based on an identifiable structure within the liver. **Some patients had tumour involving multiple hepatic segments.

FB: Free breathing, GTV: Gross tumour volume, IQR: Interquartile range, MANIV-BH: Mechanically-assisted non-invasive ventilation for breath hold.

### 3.1 Image quality assessment

**Fig. 2:**
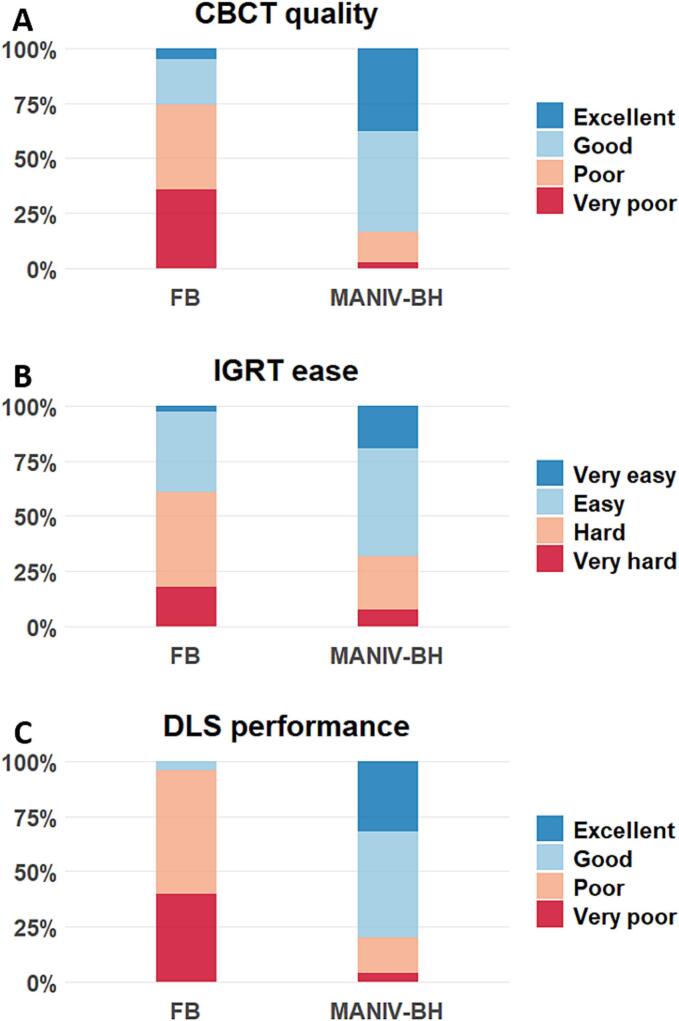
Comparison between CBCTs acquired using FB or MANIV-BH by four-point scoring scales for (A) the quality of the CBCT, (B) the ease to perform an IGRT registration between the planning CT and the CBCT, and (C) the performance of the auto-segmentation of organs-at-risk using a deep learning algorithm. (Should NOT be printed in colours) CBCT: Cone-beam computed tomography, DLS: Deep-learning segmentation, FB: Free breathing, IGRT: Image-guided radiotherapy, MANIV-BH: Mechanically-assisted non-invasive ventilation for breath hold.

Operator evaluations revealed that the quality of MANIV-BH CBCTs was significantly better than that of FB CBCTs. Specifically, 83.4 % of MANIV-BH CBCTs were rated as “Excellent” or “Good,” compared to only 25.4 % of FB CBCTs (p < 0.001, Figs. 1B and 2A).

### 3.2 IGRT variability

**Fig. 3:**
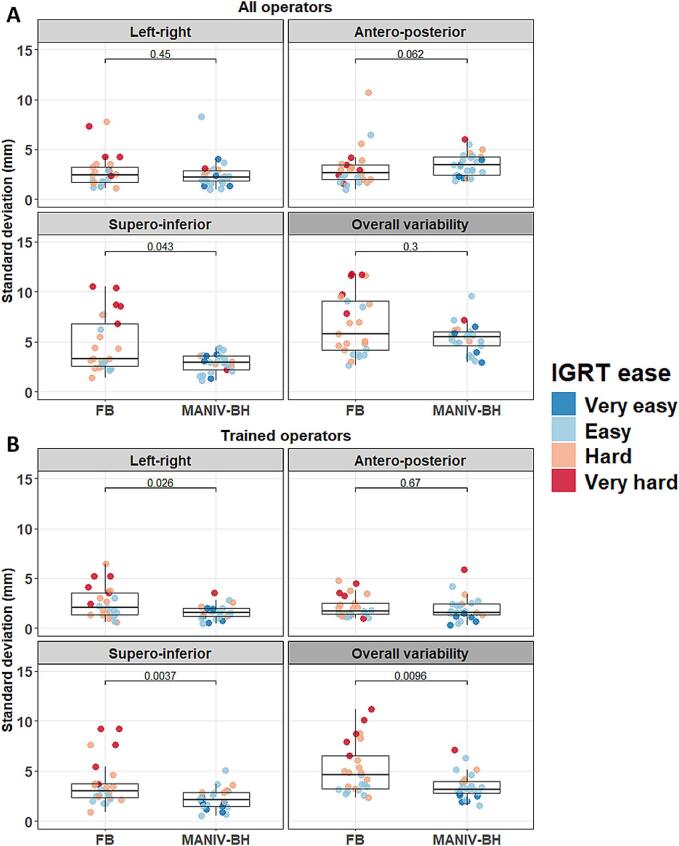
Comparison of the IGRT registration variability between CBCTs acquired using FB or MANIV-BH. Each dot represents one CBCT for which the standard deviation of the registration vector was computed among all operators. (A) In the first analysis, the IGRT performed the nine ROs and the five RTT was considered, (B) while in the second only the five ROs a sufficient training in liver SABR IGRT were included. The colours of the points, each representing a CBCT, were determined based on the most frequent IGRT ease score reported by the operators. (Should NOT be printed in colours) CBCT: Cone-beam computed tomography, FB: Free breathing, IGRT: Image-guided radiotherapy, MANIV-BH: Mechanically-assisted non-invasive ventilation for breath hold, RO: Radiation oncologist, RTT: Radiation therapist, SABR: Stereotactic ablative radiotherapy.

The ease of registration between the planning CT and the CBCT was significantly greater with MANIV-BH CBCTs (Very easy or easy: 68.0 %) compared to FB CBCTs (Very easy or easy: 38.9 %, p < 0.001, Fig. 2B). When considering all operators, the IGRT variability was similar between FB and MANIV-BH CBCTs in the left–right axis (median: 2.5 mm versus 2.3 mm, p = 0.45), the antero-posterior axis (2.6 mm versus 3.5 mm, p = 0.062), and overall (median: 5.8 mm versus 5.5 mm, p = 0.30). However, in the supero-inferior direction, the IGRT variability was significantly reduced with MANIV-BH CBCTs (median: 3.0 mm) compared to FB CBCTs (median: 3.3 mm, p = 0.043, Fig. 3A). Focusing on data from the five ROs trained in liver SABR IGRT, the IGRT variability was lower with MANIV-BH compared to FB CBCTs in the left–right direction (median: 1.6 mm versus 2.1 mm, p = 0.026), the supero-inferior direction (median: 2.2 mm versus 3.0 mm, p = 0.004), and overall (median: 3.2 mm versus 4.6 mm, p = 0.010), but not in the antero-posterior direction (median: 1.6 mm versus 1.7 mm, p = 0.67, Fig. 3B). A comparison between trained and non-trained operators revealed significantly higher IGRT variability for non-trained operators in all directions when using MANIV-BH CBCTs (p < 0.05). In contrast, for FB CBCTs, this difference was only observed in the anteroposterior direction (Supplementary Fig. 1).

### 3.3 Automatic GTV mapping

**Fig. 4:**
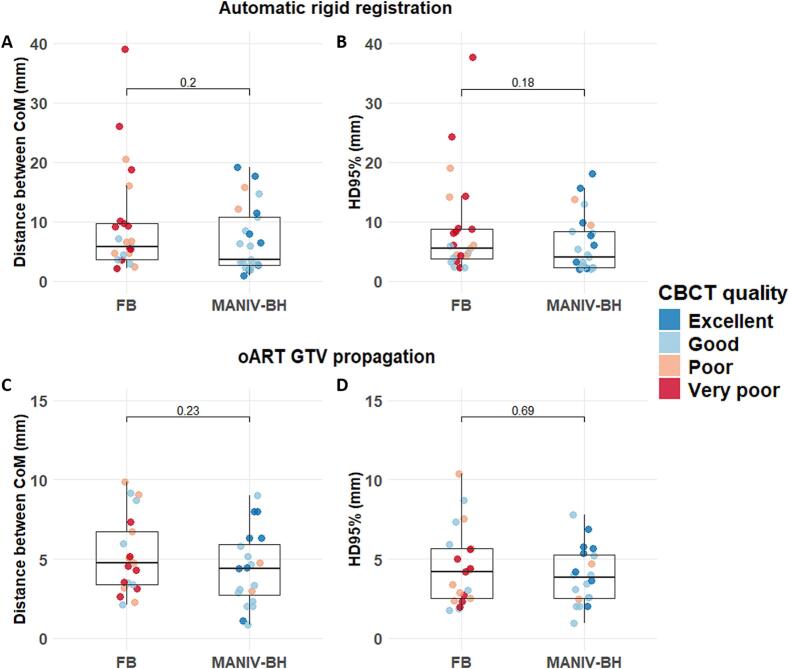
Accuracy of the automatic GTV mapping compared between FB and MANIV-BH CBCTs. The GTV propagated is compared to the median GTV position of the five trained operators that performed the IGRT registration when using (A and B) automatic rigid registration using only translation or (C and D) oART GTV propagation from the planning CT to the CBCT using an image guided deformable registration. The metrics used for the comparison of these two volumes were (A and C) the distance between the CoM (mm) and (B and D) the HD95% (mm). The colours of the points, each representing a CBCT, were determined based on the most frequent CBCT quality score reported by the operators. (Should NOT be printed in colours) CoM: Centre of mass, FB: Free breathing, GTV: Gross tumour volume, HD95: 95% Hausdorff distance, IGRT: Image-guided radiotherapy, MANIV-BH: Mechanically-assisted non-invasive ventilation for breath hold, oART: Online-adaptive radiotherapy.

For the automatic rigid registration, the median distance between the CoM of GTV_Non-ART_ and GTV_Groundtruth_ was 5.7 mm (IQR: 3.7–9.7) and 3.6 mm (IQR: 2.7–10.7) for FB and MANIV-BH respectively (p = 0.20, Fig. 4A). The median Hausdorff 95 % distance between these two structures was 5.6 mm (IQR: 3.7–8.8) for FB and 4.0 mm (IQR: 2.3–8.4) for MANIV-BH (p = 0.18, Fig. 4B).

Using oART GTV propagation based on an image-guided deformable registration, the median CoM distance between GTV_oART_ and GTV_Groundtruth_ was 4.7 mm (IQR: 3.4–6.7) for FB and 4.4 mm (IQR: 2.6–5.9) for MANIV-BH (p = 0.23, Fig. 4C). The median Hausdorff 95 % was 4.2 mm (IQR: 2.5–5.7) for FB and 3.8 mm (IQR: 2.5–5.3) for MANIV-BH (p = 0.69, Fig. 4D).

### 3.4 Deep-learning auto-segmentation performance

Once again, CBCT acquisition using MANIV-BH demonstrated superior results compared to FB when evaluating the performance of the deep-learning auto-segmentation algorithm (Excellent or good: 80.0 % versus 4.0 %, p < 0.001, Figs. 1C and 2C). Detailed results for individual OARs are provided in Supplementary Fig. 2. The median time required for manual correction of the deep-learning-generated OAR volumes was significantly reduced by 54.2 % when using MANIV-BH CBCTs (8.7 min [IQR: 4.0–11.7]) compared to FB CBCTs (19.0 min [IQR: 15.8–22.7], p < 0.001).

## 4 Discussion

This study comparing liver SABR CBCTs acquired under FB and MANIV-BH conditions highlights the clinical advantages of MANIV-BH. MANIV-BH significantly enhances CBCT image quality, facilitates IGRT, reduces interoperator IGRT variability, improves OARs auto-segmentation performance, and decreases the time required for manual edition of these volumes. These improvements are critical for accurate liver SABR delivery, with or without oART. Since MANIV-BH for liver SABR has become routine in our RT department, this study presents the first real-world results of MANIV-BH.

Accurate IGRT is essential to prevent geometric misses during liver SABR [21]. Percutaneously implanted radiopaque markers can serve as tumour position surrogates and registration targets between planning CT and CBCT [14,22]. They are more accurate than anatomical surrogates such as the diaphragmatic dome [23,24]. However, their use can be limited by patient factors, tumour location, and risks such as bleeding, pain, infection, or tumour seeding [25,26]. Also, they may cause artefacts on follow-up imaging, making their interpretation difficult. MR-guided RT offers another alternative, enabling direct tumour visualisation, smaller PTV margins, and beam interruption when the tumour moves outside the PTV [[11], [12], [13],^27^]. However, MR-guided RT is resource-intensive and not widely available [28]. While still uncommon, MANIV-BH is more accessible and less expensive than MR-linacs, making it a promising option.

Beyond the subjective benefits of improved image quality and easier registration, this study shows that MANIV-BH reduces registration uncertainties among trained operators. These findings align with previous lung SABR studies, where respiratory motion-mitigating techniques improved CBCT image quality and reduced interoperator IGRT variability [[29], [30], [31]]. Minimising IGRT variability remains crucial for tumour control and toxicity reduction. Strategies include optimising CBCT reconstruction, using modern imaging systems, and advanced IGRT training [31]. In this study, IGRT variability was significantly higher among non-trained operators, particularly for MANIV-BH CBCTs (Supplementary Fig. 1), underscoring the need for adequate training in such high-precision treatments, as demonstrated in other RT contexts [32,33].

During CBCT-guided oART, manually editing auto-segmented OAR volumes is among the most time-consuming steps. Previous *in silico* CBCT-guided oART studies reported average editing times of 24.0 min for locally advanced pancreatic cancer (within 36.3-minute sessions) and 11.4 min for abdominal metastases (22.6-minute sessions) [34,35]. In clinical treatments of gastric lymphoma, Zhong et al. reported an average session duration of 25.0 ± 6.8 min, with OAR and target volume refinement taking up most of the time [36]. Gardner et al. highlighted that improved CBCT quality reduces interoperator variability in OAR delineation, potentially lowering correction time during oART, though this remains to be confirmed [37]. Our study demonstrates that higher CBCT quality not only improves OAR auto-segmentation but may halve correction time (8.7 versus 19.0 min). This reduction offers several benefits: shorter oART sessions, reduced staffing needs, fewer intrafraction changes, and potential improvements in outcomes like lower toxicity risk [[38], [39], [40]]. Additionally, shorter sessions may enhance patient comfort and offer economic advantages.

Unlike passive respiratory motion management techniques, MANIV enables stable apneas [[17], [18], [19], [20],^41^], reducing breathing-related tumour motion uncertainties and allowing PTV margins reduction [19,42]. Other non-invasive ventilation modes, like shallow-controlled ventilation (high rate with low amplitude), also reduce PTV margins [18,43]. However, only MANIV-BH eliminates respiratory motion artefacts during CBCT acquisition, which is not possible with continuous-breathing techniques. Deep, stable inspiration apneas also provide dosimetric advantages by reducing OARs dose, and improve interfraction positional reproducibility compared to voluntary breath-hold [20]. Practical limitations include longer RT sessions (25–40 min), though comparable or shorter than gating or tracking techniques [44]. It also requires expertise and well-trained team [19,20]. We are actively developing solutions to support wider adoption of this technique.

This study has some limitations, mainly due to its *in silico* design, particularly for IGRT registration and manual correction of auto-segmented OAR volumes. Ideally, these tasks should have been performed using the Ethos® treatment platform to accurately reflect our clinical practice. However, the Ethos® emulator – which allows for the virtual delivery of both non-adaptive and oART sessions – was not available at the time of the study. Consequently, IGRT registration and OAR auto-segmentation tasks were performed using treatment planning systems different from those used clinically, and without time constraints inherent to real-life RT sessions with a patient on the couch. Therefore, absolute IGRT variability and OAR correction time values should be interpreted cautiously, while the relative benefits of MANIV-BH over FB CBCTs remain evident. Lastly, for automatic GTV propagation, the FB group compared to MANIV-BH differed from the FB group used in other analyses.

## 5 Conclusion

CBCTs acquired using MANIV-BH demonstrated superior quality compared to those obtained under FB conditions during liver SABR. High-quality CBCTs offer key advantages in conventional RT by improving IGRT precision, reducing interoperator variability for trained staff (ROs and RTTs), and making the process more comfortable. MANVI-BH also shows promise for CBCT-guided oART, as it improves the performance of deep-learning-based automatic delineation of OARs and reduces the time required for manual corrections of these volumes. Beyond the benefits of CBCT quality improvement, MANIV-BH also offers the advantage of inducing stable apneas during irradiation, which reduces tumour motion uncertainties and allows for PTV margin reductions. These advantages should further encourage teams to work on the current limitations of the MANIV-BH technique, enhance its dissemination and continue improving it, particularly in terms of dedicated time.

## Funding statement

Julien Pierrard was funded by Fonds De La Recherche Scientifique–FNRS, grant number FC 50079 and by Varian, a Siemens Healthineers Company.

## Declaration of competing interest

The authors declare the following financial interests/personal relationships which may be considered as potential competing interests: Julien Pierrard: PhD project was partially funded by Varian, a Siemens Healthineers Company. Nicolas Audag, Christel Abdel Massih, Maria Alvear Garcia, Enrique Alvarez Moreno, Andrea Colot, Simon Jardinet, Romain Mony, Ana Francisca Nevez Marques, Lola Servaes, Thaïs Tison, Valentin Van den Bossche, Aniko Wale Etume, Lamyae Zouheir, Geneviève Van Ooteghem: None..
