# Supplementary material for: Mechanically assisted non-invasive ventilation for liver SABR: Improve CBCT, treat more accurately
Source: Clin Transl Radiat Oncol. 2025 May 22;53:100983. doi: 10.1016/j.ctro.2025.100983 (PMC12163337; doi:10.1016/j.ctro.2025.100983)
Supplement: Supplementary Data 1 [file mmc1.pdf]

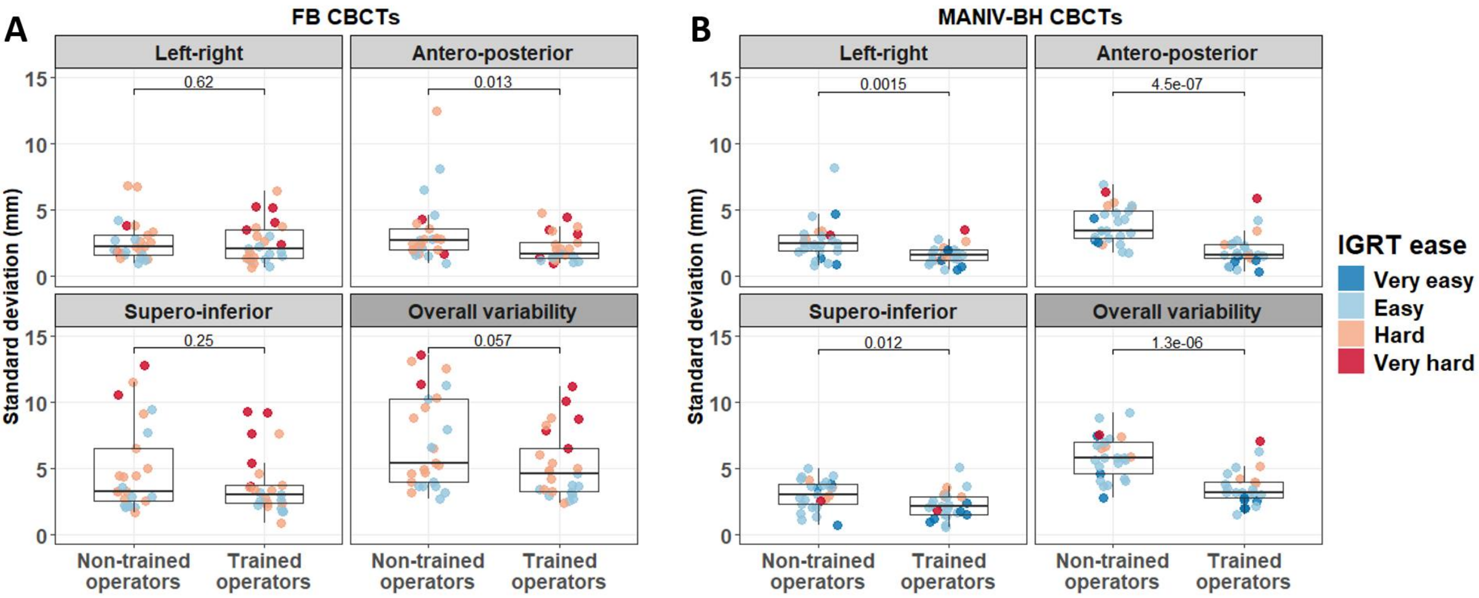


**Supplementary Figure 1 :** Comparison of the IGRT variability between non-trained operators and trained operators for (A) FB and (B) MANIV-BH CBCTs. The colours of the points, each representing a CBCT, were determined based on the most frequent IGRT ease score reported by the operators.

CBCT: Cone-beam computed tomography, FB: Free breathing, IGRT: Image-guided radiotherapy, MANIV-BH: Mechanically-assisted non-invasive ventilation for breath hold.

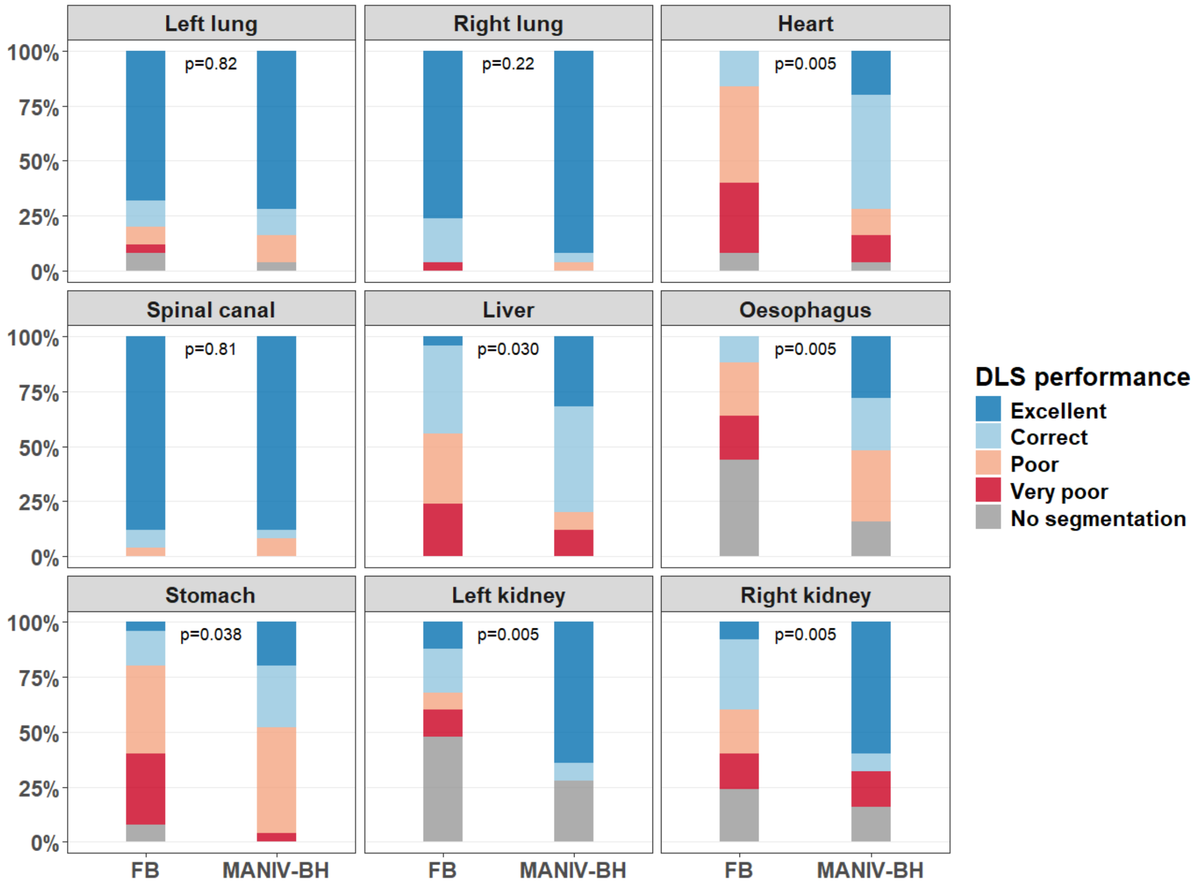


**Supplementary Figure 2:** Per organ comparison between FB and MANIV-BH CBCTs of the auto-segmentation performance of organs-at-risk using a deep learning algorithm. Statistical comparison was performed using chi-square test with Benjamini-Hochberg adjustment for multiple comparisons

CBCT: Cone-beam computed tomography, FB: Free breathing, MANIV-BH: Mechanically-assisted non-invasive ventilation for breath hold.
