# Supplementary material for: Mechanically assisted non-invasive ventilation for liver SABR: Improve CBCT, treat more accurately
Source: Clin Transl Radiat Oncol. 2025 May 22;53:100983. doi: 10.1016/j.ctro.2025.100983 (PMC12163337; doi:10.1016/j.ctro.2025.100983)
Supplement: Supplementary Data 2 [file mmc2.pdf]

	FB patients included in the “Target volume propagation accuracy” analysis
**N**	21
**Gender**	
Male	14
Female	7
**Age (median [IQR])**	65 (59 -75)
**Primary disease**	
Colorectal adenocarcinoma	14
Hepatocarcinoma	4
Ovary cancer	1
Non-small cell lung cancer	1
Breast cancer	1
**Tumour location***	
Segment I	5
Segment II	3
Segment III	1
Segment IV	5
Segment V	1
Segment VI	1
Segment VII	6
Segment VIII	8
**GTV volume (cm^3, median [IQR])**	25.9 (11.1 – 44.2)

Supplementary Table 1: Characteristics of the patients included in the “Target volume propagation accuracy” analysis.
*Some patients had tumour involving multiple hepatic segments.
FB: Free breathing, GTV: Gross tumour volume, IQR: Interquartile range.
